# Editorial

**Published:** 1882-11

**Authors:** 


### EDITORIAL.

"UNSAVORY JOURNALISM" is the title of an editorial in the November number of the *Hahnemannian Monthly*, which, we think, is a just criticism on the first article in our August issue, entitled "Homeopathy." An apology is hardly admissible; but we gave in our September issue, at the bottom of page 549, our reason for its appearance. We hope that Dr. Farrington will set us right with his readers.

